# The end of the decline in cervical cancer mortality in Spain: trends across the period 1981–2012

**DOI:** 10.1186/s12885-015-1306-x

**Published:** 2015-04-15

**Authors:** Marta Cervantes-Amat, Gonzalo López-Abente, Nuria Aragonés, Marina Pollán, Roberto Pastor-Barriuso, Beatriz Pérez-Gómez

**Affiliations:** 1 Consortium for Biomedical Research in Epidemiology & Public Health (CIBER en Epidemiología y Salud Pública - CIBERESP), Avda Monforte de Lemos 5, 28029 Madrid, Spain; 2 Cancer and Environmental Epidemiology Unit, National Centre for Epidemiology, Carlos III Institute of Health, Avda Monforte de Lemos 5, 28029 Madrid, Spain; 3 Puerta de Hierro Biomedical Research Institute, C/ Joaquín Rodrigo, 2, 28222 Majadahonda, Spain

**Keywords:** Uterine cervical neoplasms, Mortality rate, Spain, Trends

## Abstract

**Background:**

In Spain, cervical cancer prevention is based on opportunistic screening, due to the disease’s traditionally low incidence and mortality rates. Changes in sexual behaviour, tourism and migration have, however, modified the probability of exposure to human papilloma virus among Spaniards. This study thus sought to evaluate recent cervical cancer mortality trends in Spain.

**Methods:**

We used annual female population figures and individual records of deaths certified as cancer of cervix, reclassifying deaths recorded as unspecified uterine cancer to correct coding quality problems. Joinpoint models were fitted to estimate change points in trends, as well as the annual (APC) and average annual percentage change. Log-linear Poisson models were also used to study age-period-cohort effects on mortality trends and their change points.

**Results:**

1981 marked the beginning of a decline in cervical cancer mortality (APC_1981–2003_: −3.2; 95% CI:-3.4;-3.0) that ended in 2003, with rates reaching a plateau in the last decade (APC_2003–2012_: 0.1; 95% CI:-0.9; 1.2). This trend, which was observable among women aged 45–46 years (APC_2003–2012_: 1.4; 95% CI:-0.1;2.9) and over 65 years (APC_2003–2012_: −0.1; 95% CI:-1.9;1.7), was clearest in Spain’s Mediterranean and Southern regions.

**Conclusions:**

The positive influence of opportunistic screening is not strong enough to further reduce cervical cancer mortality rates in the country. Our results suggest that the Spanish Health Authorities should reform current prevention programmes and surveillance strategies in order to confront the challenges posed by cervical cancer.

**Electronic supplementary material:**

The online version of this article (doi:10.1186/s12885-015-1306-x) contains supplementary material, which is available to authorized users.

## Background

Cervical cancer is one of the most frequent female tumours world-wide, ranking second in incidence and fourth in mortality [1,2]. Rates vary widely depending on: a) the prevalence of the human papilloma virus (HPV) infection that causes this neoplasm [3]; and, b) access to and effectiveness of programmes for the early diagnosis and treatment of precancerous lesions, which can reduce the incidence of invasive cervical cancer in screened groups by approximately 80% [4].

In Spain, cervical cancer mortality rates used to be among the lowest in Europe [5]. However, the social changes experienced since the 1980’s –in the form of more liberal sexual behaviour and increased contact with people from regions with higher prevalence of infection [6,7]- have increased the risk of exposure to HPV among Spanish females in general, and among the younger cohorts in particular. Furthermore, the Spanish National Health Service’s fast pace of growth and decentralisation has modified both the coverage and quality of opportunistic cervical cancer screening. These factors, which may well have affected the epidemiology of cervical cancer, taken together with the recent incorporation of HPV vaccination strategies render it necessary for the pertinent tumour burden status to be updated in Spain, so as to be able to assess the future impact of preventive measures.

The study of cervical cancer mortality has always been hampered by the widely known phenomenon of under-registration in the certification of this cause of death [8]. In Spain, deaths coded as not otherwise specified sites of the uterus (U-NOS) represented almost 70% of all uterine cancer deaths in the early 1980s, and less than 25% since 2000. This gradual improvement in data quality, which has not been taken into account in the most recent study on cervical cancer mortality in Spain [9], directly affects and distorts time trends. To avoid the bias flowing from these changes, we reclassified U-NOS in accordance with IARC strategy [10], to analyse trends in cervical cancer mortality in Spain across the period 1981–2012, both overall and by age group and region, and fitted age-period-cohort models by incorporating a novel approach that enables possible change-points in cohort or period effects to be estimated.

## Methods

Data on mid-year population and individual death records for the period 1981–2012 were obtained from the National Statistics Institute [11]. We selected all female deaths registered as cervical cancer (ICD-9:180; ICD-10:C53), cancer of the corpus uteri (ICD-9:182.0; ICD-10:C54) and U-NOS (ICD-9:-179; ICD-10:C55), broken down by 5-year age-groups (0–4, …, 80–84 and ≥85 years).

### Reallocation of U-NOS

U-NOS deaths were reallocated to either cervical or corpus uteri cancer in line with the strategy adopted by Loos *et al*. [10]. They defined 5 age groups (0–39, 40–49, 50–59, 60–69 and ≥70 years), and quantified the annual age-specific proportion of cases registered as cervical cancer among all uterine cancer deaths, excluding U-NOS; these proportions were then applied to U-NOS to estimate cervical cancer deaths. In any case where U-NOS represented more than 25% of all uterine cancer deaths, these authors recommended the use of an external “reference population” having high data quality. In line with this criterion, Loos *et al.* applied age-specific proportions from Dutch mortality data to correct Spanish figures until 1999. Although we followed this suggestion for the period 1981–1999, we used Spanish data to compute age-specific proportions for the period 2000–2012 because U-NOS represented less than 25% of all uterine cancer deaths from 2000 onwards. As the selection of the external population is arbitrary, we evaluated the variability in our estimates by means of different approaches, namely, by applying: a) Dutch data-based proportions for the whole period; and b) Spanish proportions for the entire period (Additional file 1: Table S1).

### Age-standardised rates and Joinpoint regression analysis

We calculated crude and age-standardised mortality rates (European standard population) for each five-year period (from 1981–1986 to 2006–2010) by Autonomous Region (*Comunidad Autónoma*) (Andalusia; Aragon; Asturias; Balearic Islands; Canary Islands; Cantabria; Castile-La Mancha; Castile & Leon; Catalonia; Valencian Region; Extremadura; Galicia; Madrid; Murcia; Navarre; Basque Country; La Rioja; Ceuta and Melilla), and also computed truncated age-standardised rates for the following age groups, namely, 0–19, 20–44, 45–64 and > =65 years. Additionally, annual age-standardised mortality rates and their corresponding standard errors were calculated to study time trends. We used the NCI-Joinpoint regression analysis programme [12] to evaluate the presence of change points and estimate the annual percentage change (APC) and average annual percentage changes (AAPC), which are regarded as useful summary measurements even in cases where models may indicate the presence of changes in trend during the study period [12].

### Age–period–cohort models

Log-linear Poisson models were fitted to study the effect of age, period of death and birth cohort on mortality trends. For this purpose, five-year age-groups and quinquennia for the period 1981–2010 were used, excluding the open-ended category of persons aged over 85 years and women aged <20 years, due to the limited number of deaths. To overcome the problem of non-identifiability of model parameters arising from exact linear dependence among age, period, and cohort [13], we adopted the approach proposed by Holford [14], and considered estimable functions of parameters, such as the curvatures in each effect and the sum of period and cohort linear slopes, also known as net drift. We estimated effects curvature and net-drift, which are uniquely determined by the data and hence remain invariant irrespective of the particular approach used [15], and displayed the cohort and period effects graphically. We also checked for extra-Poisson dispersion [16].

### Curvature change points

To detect changes in the period and cohort effects of the three-factor model, separate Joinpoint regression analyses of the estimated period and cohort curvatures were performed with weights inversely proportional to their estimated variances [17]. These models provided the number of significant change-points across periods and cohorts by using permutation tests, their estimated locations and the associated changes in slopes.

## Results

From 1981 to 2012, a total of 16,669 deaths were originally certified as cervical cancer deaths in Spain. After reallocating U-NOS deaths, however, the number of estimated deaths due to this tumour rose to 26,699. The original and corrected figures for cervical cancer deaths, as well as the distribution of U-NOS by period, age group and region can be evaluated in Table 1.

**Table 1:** **Cervical cancer deaths (original and corrected): Spain,1981-2012**

	Cervical cancer deaths (C53)		All uterine cancer deaths (C53-C55)	U-NOS^a^deaths (C55)		
	Original	Corrected	*N*	N	% of all deaths	% recoded as cervical cancer (C53)
	*N*	*N*				
**Total**	16669	26699	59899	22762	38%	44%
**Period**						
1981-1985	1760	4954	9400	6392	68%	50%
1986-1990	2314	4611	9082	4813	53%	47%
1991-1995	2624	4201	9038	3525	39%	45%
1996-2000	2749	3783	8928	2500	28%	41%
2001-2005	2741	3526	9000	2160	24%	36%
2006-2010	3139	3953	9992	2198	22%	37%
2011-2012	1342	1669	4459	936	21%	35%
**Age group**						
<45 years	2541	3339	3704	926	25%	88%
45 to 64 years	6471	10081	17279	6220	36%	57%
> = 65 years	7657	13279	38916	15566	40%	37%
**Geographical area**						
***Northern region***						
Galicia	1401	2368	4982	2142	43%	45%
Asturias	605	921	2173	717	33%	44%
Cantabria	243	360	860	267	31%	44%
Basque Country	710	1160	2852	1027	36%	44%
Navarre	131	248	719	288	40%	41%
***Central Region***						
Castile & Leon	852	1497	3647	1459	40%	44%
Castile-La Mancha	557	1035	2560	1075	42%	44%
Extremadura	274	583	1462	702	48%	44%
Madrid	1906	2728	6011	1863	31%	45%
Aragon	369	746	1975	869	44%	43%
La Rioja	77	144	395	150	38%	45%
***Mediterranean & Southern region***						
Catalonia	2776	4254	9612	3364	35%	44%
Valencian Region	1765	2897	6532	2547	39%	44%
Murcia	438	718	1624	617	38%	45%
Andalusia	2963	4892	10402	4265	41%	45%
***Balearic & Canary Islands***						
Balearic Islands	546	769	1465	498	34%	45%
Canary Islands	976	1260	2432	632	26%	45%
***Autonomous City enclaves (North Africa*** **)**						
Ceuta	40	61	101	45	45%	47%
Melilla	40	58	95	32	34%	55%

^a^NOS: not otherwise specified.

**Figure 1:**
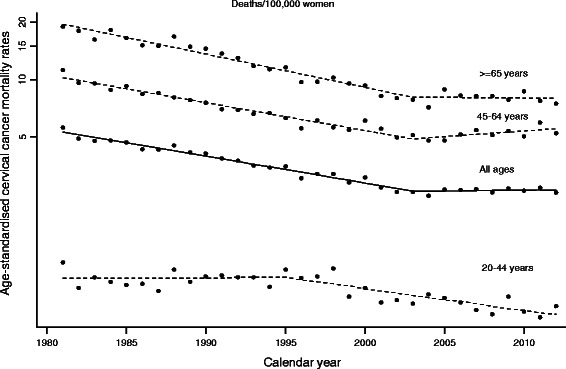
Cervical cancer mortality in Spain (1981–2012). Observed age-standardised rates and estimated trends for women, both overall and by age group. Points: observed age-standardised rates. Lines (dashed and solid): modelled age-standardised rates (modelled data results from Joinpoint).

Figure 1 depicts age-adjusted cervical cancer mortality rates for the whole period, overall and by age-group, with a detailed breakdown being shown in Table 2.

**Table 2:** **Age-standardised cervical cancer mortality rates (deaths per 100,000 women): trends in Spain,1981-2012** ^**+**^

	% women >15yrs never screened^++^		Age-standardised mortality rates (100,000 women-year)								Average annual percentage change		Annual percentage change									
		Deaths	1981	1986	1991	1996	2001	2006	2011	1981	1981-2012		First period		Change point		Second period		Change point		Third period	
		*N*	1985	1990	1995	2000	2005	2010	2012	2012	%	(95% CI)	%	(95% CI)	Year	(95% CI)	%	(95% CI)	Year	(95% CI)	%	(95% CI)
**Total**	27	26699	4.9	4.3	3.6	3.1	2.6	2.6	2.6	3.4	**−2.2**	**(−2.6;-1.9)**	**−3.2**	**(−3.4;-3.0)**	2003	(2001–05)	0.1	(−0.9;1.2)				
Age group*																						
20-44 years	28	3328	1.6	1.6	1.7	1.6	1.3	1.2	1.1	1.4	**−1.4**	**(−2.2;-0.6)**	0.1	(−1.2;1.5)	1995	(1990–00)	**−2.6**	**(−3.6;-1.7)**				
45–64 years	14	10081	9.7	8.1	6.7	5.8	5.0	5.2	5.6	6.6	**−2.0**	**(−2.4;-1.5)**	**−3.3**	**(−3.7;-3.0)**	2003	(2000–06)	1.4	(−0.1;2.9)				
≥65 years	43	13279	17.6	15.3	12.3	9.7	8.0	8.2	7.6	11.0	**−2.9**	**(−3.4;-2.3)**	**−4.0**	**(−4.4;-3.5)**	2003	(2000–05)	−0.1	(−1.9;1.7)				
**Geographical area**																						
***Northern Region***																						
Galicia	40	2368	5.4	5.3	4.1	4.0	3.2	2.9	3.4	4.0	**−2.4**	**(−2.8;-1.9)**										
Asturias	30	921	4.9	4.8	4.7	3.7	2.3	3.0	2.5	3.8	**−2.6**	**(−3.4;-1.7)**										
Cantabria	41	360	4.5	4.4	3.7	3.1	2.8	2.5	2.6	3.4	**−2.4**	**(−3.4;-1.4)**										
Basque Country	19	1160	3.9	3.4	2.7	2.3	2.3	2.4	2.0	2.7	**−2.2**	**(−2.8;-1.5)**										
Navarre	25	248	2.3	3.3	2.1	1.5	1.9	1.8	2.0	2.1	**−1.9**	**(−3.2;-0.5)**										
***Central Region***																						
Castile & Leon	27	1497	3.9	3.4	2.6	2.7	2.3	2.1	2.5	2.8	**−2.2**	**(−2.8;-1.6)**										
Castile-La Mancha	33	1035	4.6	3.3	2.8	2.4	2.1	2.6	2.4	2.9	**−1.7**	**(−3.1;-0.3)**	**−3.9**	**(−5.0;-2.7)**	2002	(1991–05)	3.1	(−0.9;7.2)				
Extremadura	39	583	4.4	3.0	3.0	2.2	2.2	2.1	1.9	2.7	**−2.7**	**(−3.5;-1.8)**										
Madrid	21	2728	3.3	3.4	3.1	2.7	2.3	2.1	2.2	2.7	**−2.0**	**(−2.5;-1.4)**										
Aragon	31	746	4.0	3.2	3.3	2.5	2.1	2.0	2.0	2.7	**−2.7**	**(−3.5;-1.9)**										
La Rioja	23	144	4.5	2.1	2.8	1.8	1.8	2.5	3.1	2.6	−1.7	(−4.1;0.7)										
***Mediterranean & Southern Region***																						
Catalonia	20	4254	5.3	4.6	3.7	2.9	2.2	2.5	2.2	3.3	**−3.4**	**(−5.3;-1.6)**	**−4.4**	**(−4.9;-3.9)**	2004	(1984–07)	3.6	(−2.2;9.6)	2010	(2001–10)	−12.3	(−32.2;13.3)
Valencian Region	21	2897	5.7	4.5	3.6	3.4	2.9	2.8	3.2	3.6	**−2.1**	**(−3.0;-1.3)**	**−3.5**	**(−4.2;-2.8)**	2002	(1990–09)	0.8	(−1.6;3.2)				
Murcia	33	718	5.5	4.6	4.7	3.6	2.5	2.7	2.6	3.6	**−2.9**	**(−3.7;-2.1)**										
Andalusia	40	4892	6.0	4.7	4.1	3.5	3.0	2.8	2.8	3.8	**−2.5**	**(−3.2;-1.8)**	**−3.5**	**(−3.9;-3.0)**	2004	(1993–08)	0.3	(−2.2;2.9)				
***Balearic & Canary Islands***																						
Balearic Islands	22	769	6.6	7.6	6.3	3.9	4.2	3.6	3.4	5.0	**−2.9**	**(−3.8;-2.0)**										
Canary Islands	15	1260	7.1	5.6	5.1	4.3	3.7	4.0	4.1	4.7	**−2.0**	**(−2.6;-1.4)**										
***Autonomous City enclaves (North Africa)***																						
Ceuta	30	61	7.9	6.1	7.7	3.4	6.1	5.5	2.2	5.8												
Melilla	58	58	5.8	7.4	6.2	9.1	4.7	5.9	2.8	6.2												

*Age group 0–19 years: total number of cases for the whole period: 11 cases; rates not shown.

^+^Joinpoint regression trend analyses by age group and geographical region.

Bold text: Statistically significant annual percentage change. ^++^% women aged >15 years reporting no citology screening test (Pap smear) in their lives by 2012 (National Health Surveys).

Cervical cancer mortality experienced a marked decrease (AAPC: −2.2%; 95% CI −2.6; -1.9) but underwent two different phases: a) an initial period (1981–2003), with rates decreasing by −3.2% per annum (95% CI: −3.4; -3.0); and b) a second period, from 2003 to the end of the study period, with stable rates (APC: 0.1%; 95% CI: −0.9; 1.2). A breakdown by age group showed that both middle-aged (45–64 years) and older women (≥65 years) registered similar trends, with mortality clearly declining until 2003 (APC_45–64_:-3.3; 95% CI:-3.7;-3.0; APC_≥65_:-4.0; 95% CI:-4.4;-3.5) and rates levelling-off thereafter. In the younger groups (20–44 years), in contrast, cervical cancer mortality was initially stable but from 1995 onwards rates began to decrease by around −2.6% per annum (95% CI: −3.6; −1.7).

Time trends by geographical area also reflect this same pattern, i.e., cervical cancer mortality decreased in all regions, with average annual percentage changes ranging from −1.7 (Castile-La Mancha) to −3.4 (Catalonia). However, recent trends in the Mediterranean and Southern region merit special attention: whereas their high mortality rates initially experienced a steep fall of around −4% per annum, in recent years these trends have changed, with mortality remaining stable in the Valencian Region (APC: 0.8; 95% CI:-1.6; 3.2) and Andalusia (APC: 0.3; 95% CI:-2.2; 2.9), and displaying a fairly unsteady trend in Catalonia (APC_2004–2010_: 3.6; 95% CI:-2.2; 9.6; APC_2010–2012_: −12.3; 95% CI:-32.2; 13.3). Owing to the low number of cases, Joinpoint regression models could not be fitted for the Autonomous City enclaves of Ceuta and Melilla; their mortality rates, which remain high, have almost halved in the last two years, though these changes might be due to the high variability in rates. The change in age-specific proportions did substantially not modify any of these results (Additional file 1: Table S1).

**Figure 2:**
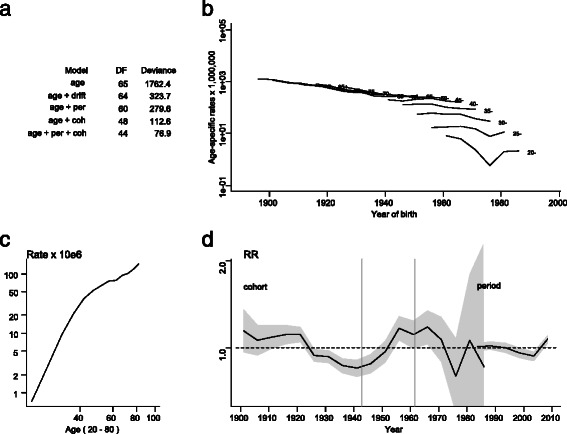
Cervical cancer mortality in Spain (1981–2010); age-period-cohort analysis. **a)** Deviance table for age-period-cohort models; **b)** Trends in age-specific rates by birth cohort; **c)** Cross-sectional age effect for an average period; **d)** Curvature of period and cohort effects and change points in cohort curvature (vertical grey lines).

Figure 2 shows the age-specific mortality rates by birth cohort and graphically depicts the results of the age-period-cohort analysis, drawn from the best-fit model which included the three components (age + period +cohort). As expected, cervical cancer mortality rates increased with age, stabilised at around the age of 60 years and then started rising again. As regards the cohort effect, our analysis identified two significant change points: dating from the beginning of the 20th century, risk declined markedly with birth cohort until the early years after the Spanish Civil War. At about this time -the early 1940’s- the probability of dying due to cervical cancer in Spanish women began to increase by birth-cohort until 1962, when the risk again moved sharply downward. The fluctuating cohort effect in the most recent generations reflects the instability of rates in these birth cohorts, solely represented in our study by women in the youngest age-groups which have a very small number of deaths. Insofar as the period effect was concerned, our results suggest a decline in risk until 2003, followed by stabilisation in the last five-year period, though there was no statistically significant change point in evidence.

## Discussion

This study describes invasive cervical cancer mortality trends in Spain over the last 32 years. If the whole period is considered, mortality rates have clearly declined; nevertheless, our report’s most relevant result is the change in trend detected in the last ten years, when rates stopped falling and reached a plateau. This trend is more evident in Spain’s Mediterranean and Southern regions, and can be observed in women both in the 45- to 65- and over 65-year age ranges. Younger women, in contrast, displayed a different temporal pattern, with stable rates until 1995 and a significant decline, of close on 3% per annum, thereafter. While mortality is the most comprehensive and homogeneous source of information on cancer in Spain, there is a clear under-registration when it comes to certification of death due to this tumour in Spain [8]. To address this problem, we adopted a simple, reproducible and widely used strategy [10,18,19], yet its clearest limitation resides in the possible non-representativeness of the selection of the reference population. Our sensitivity analyses reinforce the reliability of our results.

In most developed countries, cervical cancer mortality rates have fallen markedly since the introduction of systematic cytological screening [20]. In Spain, Joinpoint analysis shows a decline in mortality until 2003, which is line with the moderate decrease in incidence reported for the period 1980–2004 [21]. The opportunistic Spanish cervical cancer screening programme, taken together with the advances in cervical cancer treatment, probably explains the trend observed in this initial period. The country’s sociological evolution may have strengthened the impact of the preventive effect of this screening at a population level. According to our age-period-cohort models, the trend in the risk of dying due to this tumour changed among women born between 1950 and 1960: Spanish women, who had a low prevalence of HPV and low cervical cancer rates, experienced a marked transformation in their social role in the latter years of Franco’s military dictatorship and early years of democracy, and underwent major changes in their habits, including their sexual and smoking behaviours [6]. Highly educated women played a pioneering role in this process. At this time, more conservative sexual behaviours (i.e., life-long monogamy) were more usual among females with a primary or lower educational level than among those with a university education (80% vs. 50%) [6]. As such women with a higher educational level are usually more prone to attend cytological screening [7], the opportunistic strategy may have unintentionally targeted this high-risk subgroup in the latter part of the 20th century.

Recently, however, some countries, such as The Netherlands [22], USA [23] and England [24], have reported changes in the trend in mortality due to this cancer, with a slow-down in the decline in rates. In Spain, this shift in the trend has been more marked, in that mortality has even stopped declining and levelled off, indicating that the positive influence of opportunistic screening is currently not strong enough to offset increased exposure to HPV. Nowadays, the combination of the generalisation of more permissive sexual behaviours [6,25] and international tourism have increased the probability of exposure to HPV among all Spanish women. The mean age of first sexual intercourse among Spanish girls in the 1970–1980 birth cohorts is more than one year younger than that of the 1950–1960 birth cohorts [6,25], thereby favouring earlier HPV infection and more persistent cases, due to cervical immaturity. The proportion of non-sexually active females has also dropped over time, and women –as well as men- have clearly increased their lifetime number of sexual partners [6,25], facilitating the acquisition of high-risk VPH. Despite the fairly extensive population coverage of cervical screening -around 72% of Spanish women over the age of 25 years report undergoing at least one cytology screening test (Pap smear) in their lives [26]- there is still a wide gap between the proportion of never-screened women among those in higher managerial or professional positions and those employed as unskilled workers (15% vs. 36%) [26], and many women are still being diagnosed without ever having attended any screening test whatsoever [27].

The divergent trend seen in the youngest age group is extremely interesting, however. Among these women, mortality rates were stable until 1995, at which point they started to decline by 2.7% per annum. These differences among age groups have also been observed in cervical cancer incidence [21]. Even though sexual behaviours that facilitate exposure to HPV are more prevalent among young Spanish females [28], health surveys indicate that they are also more likely to report a recent Pap smear [26,29].

There was also evidence of a certain degree of heterogeneity in time trends by geographical area, with the stabilisation in mortality figures being mainly found in south-west Spain and rates in other regions still on the decline. Cervical cancer mortality in Spain is quite variable by region, with rates traditionally being lower in the more conservative areas in central Spain than in the coastal regions (Additional file 2: Figure S1). Health surveys show that women in the Mediterranean and Southern region, and on the islands, where beach tourism has for many years been one of the main economic activities, report a younger age at first sexual intercourse and a higher number of sexual partners [6]. Health policy decisions have also to be considered when studying spatial variation, as the Spanish National Health Service is heavily decentralised, with very important organisational differences in preventive protocols and coverage as between the various regions [29], i.e., in some areas, such as Asturias, La Rioja and Castile & Leon, public health screening has been reinforced and is not purely opportunistic, since part of the population is invited to attend by the health authorities [30], while in others, private health practices have a very relevant share in cervical cancer screening coverage [31]. Hence, more than 20% of women in Madrid, Catalonia and the Balearic Islands have double (public and voluntary private) health insurance coverage, as compared to the low proportion found in other areas (i.e., less than 3% in Navarre, Cantabria and Melilla) [26].

The relevance of the immigrant population in Spain warrants special attention. Since the 1990’s, Spain has become the destination for an important influx of immigrants from countries with higher rates of cervical cancer [11]. The total number of foreigners residing in Spain increased from 350,000 in 1991 to 1,600,000 in 2001, and rose to 5,250,000 in 2011 [11]. Female immigrants currently account for over 13% of women in some regions (such as the Balearic and Canary Islands, Catalonia, Valencian Region, Murcia and Madrid), and have two very different profiles: a) young women, coming mainly from South America (i.e., Ecuador, Colombia and Bolivia), Eastern Europe (Romania, Bulgaria and Poland) and North Africa (mostly Morocco), who represented 17% of all women aged 20 to 44 years residing in Spain in 2011 [11], are mainly economic immigrants and include a substantial number of unregistered residents; and b) older women, usually born in Germany or the UK, living in Mediterranean areas or on the Islands, to which they moved in middle age or on retirement. These two groups also differ in terms of screening coverage: while among older women, the proportion of never screened is higher in foreigners than in native Spaniards (48% vs. 34%), among women aged 25–64 years the opposite is true (14% vs. 27%) [32]. Recent legislation (Royal Law-Decree 16/2012) has imposed severe restrictions on health-care access for undocumented foreign residents and will probably reduce screening coverage in this subgroup of women, usually considered a high-risk group for this cancer.

## Conclusions

The decline in cervical cancer rates, a disease seen as an avoidable cause of death, has come to a halt in Spain. These data indicate that the current prevention programmes, which are based on opportunistic screening, are not capable of further reducing the rates, even though the comparison with other countries, such as Sweden and Finland, make it clear that there is still room for improvement [2].

Moreover, in the near future, screening will have to take into account the possible changes in infection dynamic derived from HPV vaccination [33], which was included in the publicly funded Spanish vaccination schedule in 2007 [25], and the availability of the HPV test [34,35]. The Spanish population-screening programme network has recently suggested changes in public cervical cancer screening [36] focused on two main points: a) a new standard screening protocol, recommending cytology for sexually-active women under the age of 35 years and high-risk HPV detection [37] among women over this age threshold, with new triage and follow-up strategies for those with positive results; and, b) the incorporation of this protocol in organised, public, population-based screening programmes, including adequate surveillance systems to assess performance. At present, specific strategies should at least prioritise subgroups of women with low screening rates, though global public health measures are needed to reform and reinforce prevention for this neoplasm, in order to face the challenges posed by cervical cancer in Spain in the 21st century. In addition, clear actions should be taken to strengthen cervical cancer surveillance: the lack of national cancer incidence registries as well as current problems in the quality of cervical cancer mortality data are equally important issues that health authorities should address.

## Additional files

Additional file 1: Table S1. Joinpoint regression trend analyses by age group and geographical region using cervical cancer proportions obtained from different reference populations.
Additional file 2: Figure S1. Age-standardised cervical cancer mortality rates in Spain by Autonomous Community (Deaths/100.000 women). European Standard Population.

## Abbreviations

APC: Annual percentage change
CI: Confidence interval
HPV: Human papilloma virus
U-NOS: Not otherwise specified sites of the uterus
AAPC: Average annual percentage change
