# Supplementary material for: The end of the decline in cervical cancer mortality in Spain: trends across the period 1981–2012
Source: BMC Cancer. 2015 Apr 15;15:287. doi: 10.1186/s12885-015-1306-x (PMC4408572; doi:10.1186/s12885-015-1306-x)

FigureS1. Age-standardised cervical cancer mortality rates in Spain by Autonomous Community (Deaths/100.000 women). European Standard Population

a) Period 1981-1985 **b) Period 2006-2012**

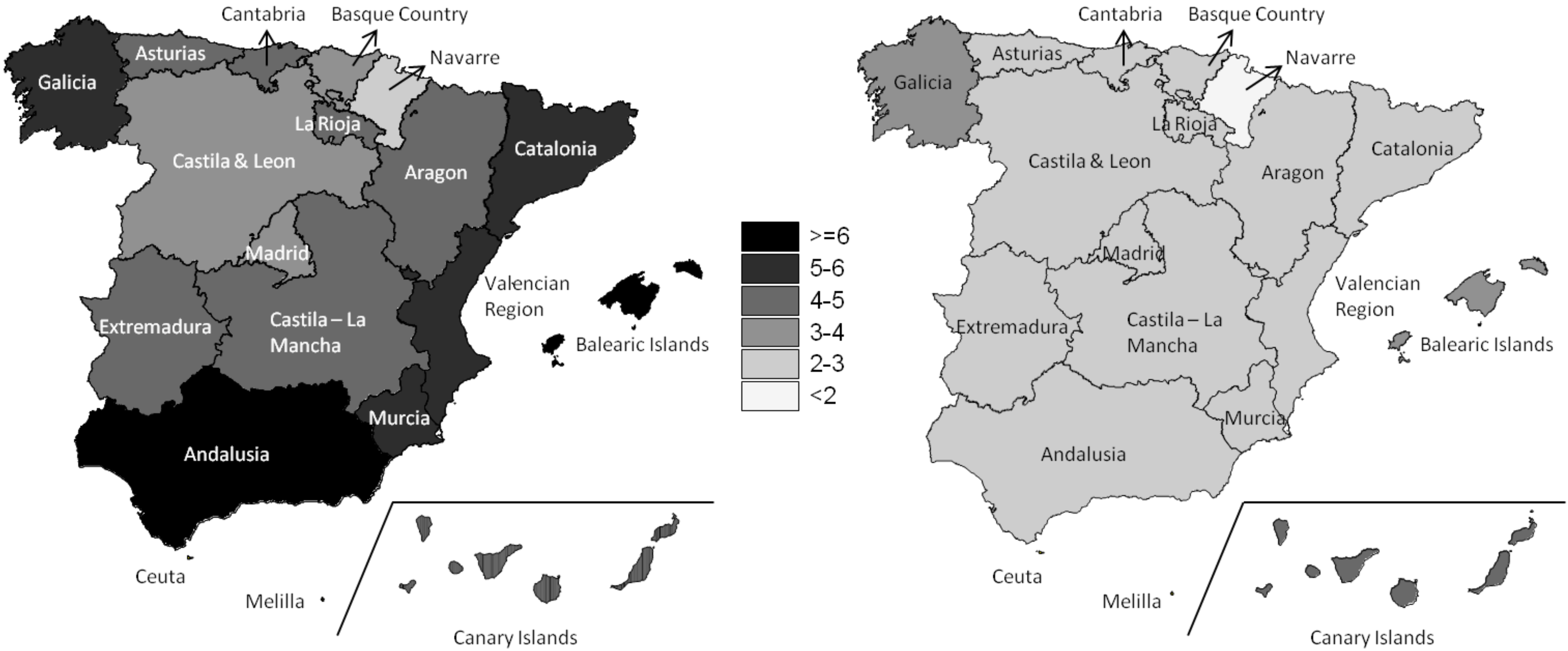

Supplement: Additional file 2: Figure S1. — Age-standardised cervical cancer mortality rates in Spain by Autonomous Community (Deaths/100.000 women). European Standard Population. [file 12885_2015_1306_MOESM2_ESM.pdf]
